# Unraveling complexity in climate change effects on beneficial plant–microbe interactions: mechanisms, resilience, and future directions

**DOI:** 10.1111/nph.70644

**Published:** 2025-10-14

**Authors:** Michelle E. Afkhami, Aimée T. Classen, Collin G. Dice, Damian J. Hernandez, Vicki W. Li, Amanda H. Rawstern, Jennifer A. Rudgers, John R. Stinchcombe, Kerri M. Crawford

**Affiliations:** ^1^ Department of Biology University of Miami Coral Gables FL 33146 USA; ^2^ Department of Ecology and Evolutionary Biology University of Michigan Ann Arbor MI 48109 USA; ^3^ Department of Biology and Biochemistry University of Houston Houston TX 77204 USA; ^4^ Department of Ecology and Evolutionary Biology University of Toronto Toronto ON M5S 3B2 Canada; ^5^ Department of Biology Sevilleta Long Term Ecological Research Program, and ARID Institute, University of New Mexico Albuquerque NM 87131 USA

**Keywords:** climate change, drought, legacy effects, microbiome, plant–microbe mutualism, resilience, salinity, stability

## Abstract

Plant microbiomes have the potential to mitigate the impacts of climate change, yet both the complexity of climate change and the complexity of plant–microbe interactions make applications and future predictions challenging. Here, we embrace this complexity, reviewing how different aspects of climate change influence beneficial plant–microbe interactions and how advances in theory, tools, and applications may improve understanding and predictability of climate change effects on plants, microbiomes, and their roles within ecosystems. New advances include consideration of (1) interactions among climate stressors, such as more variable precipitation regimes combined with warmer mean temperature; (2) mechanisms that promote the stability of microbiome functions; (3) legacies of stress affecting the functionality of microbial communities under future stress; and (4) temporally repeated plant–microbe interactions or feedbacks. We also identify key gaps in each of these areas and spotlight the need for more research bridging molecular biology and ecology to develop a more mechanistic understanding of how climate change shapes beneficial microbe–plant interactions.

**Table:** 

	Contents	
	Summary	93
I.	Introduction	94
II.	Complex climate disruptions of beneficial plant–microbe interactions	94
III.	Burgeoning advancements in climate change effects on beneficial plant‐microbial interactions	99
IV.	Conclusion	107
	Acknowledgements	109
	References	109

## I. Introduction

Climate is changing at an unprecedented rate, and ecologists are tasked with predicting the consequences for communities. Plants, which underpin terrestrial communities, host rich microbiomes that influence how plants respond to the environment. While some microbes are pathogens that negatively affect plants (see reviews on climate change effects on pathogens: Raza & Bebber, 2022; Singh *et al*., 2023; Gallego‐Tévar *et al*., 2024), many other members of microbial communities are crucial to plant growth, survival, and fecundity, with cascading effects on plant community dynamics and ecosystem functions (Porter *et al*., 2020; Duan *et al*., 2024). Importantly, beneficial microbial players, such as mycorrhizal mutualists and plant growth‐promoting bacteria, can help plants cope with the novel stressors that accompany climate change (Rudgers *et al*., 2020), and microbial communities themselves can rapidly respond to changes in climate (Castro *et al*., 2010). Therefore, microbial communities have the potential to both mitigate and amplify the consequences of climate change on plants (Trivedi *et al*., 2022).

Understanding whether and how microbial communities respond to climate change is essential to understanding how they in turn shape plant responses to climate change, and the broader ecosystem services that plants and microbes support. For example, climate change can cause local extinctions of key microbial taxa (Ochoa‐Hueso *et al*., 2018), homogenize microbial communities across landscapes (Jiajun *et al*., 2024), and decouple interactions between microbes and plants (Sorensen *et al*., 2020), leading to unanticipated and consequential changes in the outcomes of plant–microbe interactions. Scaling up, climate‐mediated changes in plant–microbe interactions can alter plant community dynamics and the functional outcome and rate of ecosystem processes.

Importantly, climate change is actually a complex mix of stressors that vary not only in type (e.g. warming and drought) but also in time, intensity, and history. The interplay between these different dimensions can complicate understanding how microbes mitigate environmental stress for plants. Here, we review how climate change influences beneficial plant–microbe interactions and highlight necessary advances in theory and techniques to improve both our understanding and predictions of how the complex interactions between different aspects of climate change coalesce to structure plant‐microbial ecology in a more stressful world.

## II. Complex climate disruptions of beneficial plant–microbe interactions

**Fig. 1:**
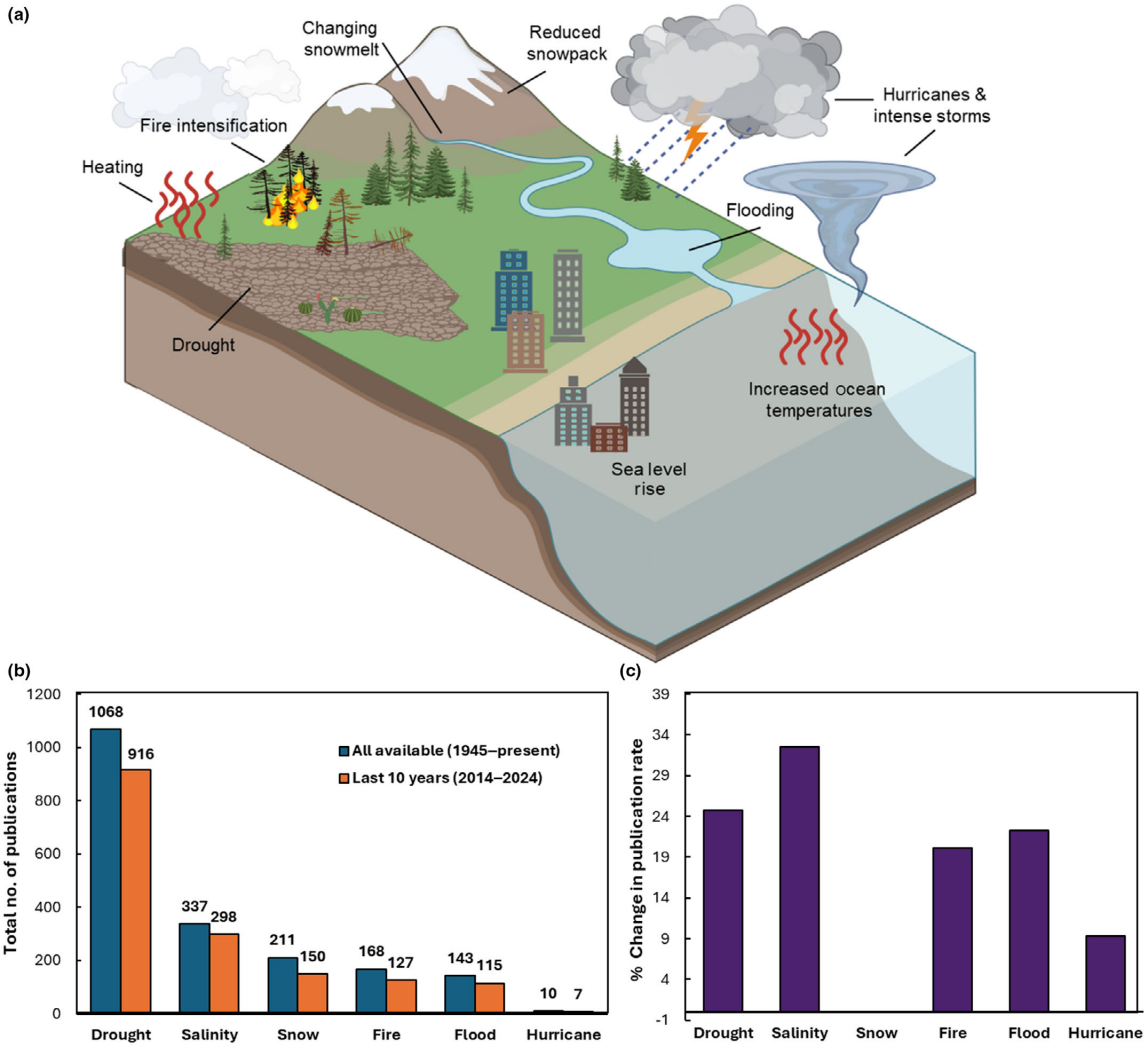
Climate change effects on our ecosystems are diverse, but research on plant–microbe interactions has focused on drought. (a) Changes in temperature and precipitation under global climate change are leading to increased drought/aridity, salinity stress from sea level rise, disruptions to snowpack and snowmelt timing, and intensification of extreme climate events, such as fires, floods, and hurricanes/storms. Created in BioRender. Afkhami, M. (2025) https://BioRender.com/gfe0em8. (b) Vote counting analysis of publications in the Web of Science database (Clarivate, 2024) showed that 55–57% addressed drought both recently (last 10 yr) and over longer time frames (1945–present). Far fewer studies investigated other aspects of climate change; in fact, there are more plant‐microbial papers referencing climate change effects of drought than all five other climate change effects combined. (c) Interestingly, the % change in publication rate over the last decade (2014–2024) showed that research into all climate factors is accelerating with the exception of snow effects, which has maintained a consistent, low level of research. The greatest acceleration of research has been for salinity, while snow shows the slowest rate of growth. (b, c) The data used in these panels were from Web of Science searches that all included plant AND microb* AND climate change, AND X, where X was the specific climate stressor. In (b) blue bars show counts from all available data (1945–present) while orange bars show data from the last decade (2014–2024). In (c) the rate of change was calculated for each climate factor across the last decade (2014–2024) by transforming the number of papers in each year for that climate factor (log_2_ transformation to improve normality), regressing the transformed paper counts on year, then calculating the % change in the publication rate per year as the (2^β^ − 1) × 100 where β is the regression coefficient. Please see the Supporting Information Methods S1, Tables S1 and S2 for more details on how this literature search was performed.

In this section, we break down climate change into types of impacts (Fig. 1), briefly summarizing the current state of knowledge for beneficial plant–microbe interactions, which provide foundational knowledge for our discussion of current areas of exploration and promising future directions in the second section.

### 1. Escalating aridity and drought

Rising global temperatures and declining precipitation are increasing aridity in many ecosystems (Calvin *et al*., 2023), and a diversity of microbes can increase plant tolerance or resistance to drought stress. For example, arbuscular mycorrhizal (AM) fungi transport water to their hosts and can contribute over 30% of plant water uptake (Kakouridis *et al*., 2022). Similarly, ectomycorrhizal fungi can redistribute water from deep soil layers to plant roots in drier surface layers through hydraulic lift (Querejeta *et al*., 2007). Ascomycota root endophytes, though relatively understudied, are common in arid and high elevation ecosystems (Jumpponen & Trappe, 1998), and can help plants resist drought through changes in plant physiology and root architecture (Malicka *et al*., 2022). Even aboveground microbes, like leaf fungal endophytes, can aid their host plants in avoiding or tolerating drought stress by affecting stomatal closure, root architecture, and osmotic adjustments (Kannadan & Rudgers, 2008). Bacteria, too, can help plants withstand drought stress through a variety of mechanisms, including phytohormone production, modification of root architecture, changes in phenology, and the maintenance of soil water capacity through biofilm production (Naylor & Coleman‐Derr, 2018).

Microbes differ in their sensitivity to heat, aridity, and drought. Fungi form hyphal networks to buffer against water limitation, while bacteria often survive through dormancy (Canarini *et al*., 2024). Some microbes produce protective osmolytes to maintain activity under drought‐induced osmotic stress (Schimel, 2018). Microbes that lack adaptations to drought may be lost from communities, and even tolerant taxa may decline in activity under drought (Potts, 1994), leading to changes in microbial community structure or function (Box 1). Some studies find that soil fungi are more resistant (i.e. have less species turnover after drought perturbation; Box 1) than bacterial communities under drought, but are less able to recover (i.e. revert back to their pre‐drought perturbation community structure and function; Box 1) than bacterial counterparts when pushed too far (de Vries *et al*., 2018; Canarini *et al*., 2024). Plants can also influence microbial responses to heat, aridity, or drought, but there is conflicting evidence about whether and how much plants buffer microbes (de Vries *et al*., 2012) or intensify drought‐generated shifts in microbial community composition (Veach *et al*., 2020). How plant carbon resources available to microbes, such as root exudates, respond to drought may explain some of this variation in buffering, as some plant species can maintain exudates under drought while others reduce exudation (Williams & de Vries, 2020).

Box 1 **Glossary of key terms**

This glossary provides definitions of bolded terms throughout the paper, focusing predominantly on ecological and microbiome terms that may be less familiar than plant‐associated terms. While some of these terms have been widely discussed in detail and with nuance in the broader literature, this glossary explains how we use these terms specifically, providing important context to welcome debate and discussion of how climate change shapes plant‐microbial interactions across biological subdisciplines.

**Alternative stable states:** Equilibrium ecosystem states with corresponding structure and function. Environmental stress and perturbations can push an ecosystem beyond a tipping point, shifting the ecosystem from its original state to an alternative state. Returning to the original state may require a greater perturbation or effort than it took to cause the initial shift to the alternative state.

**Compartmentalization:** The division of communities into relatively independent, distinct groups – called compartments – where taxa within a compartment have more frequent interactions or associations with each other than with those taxa in other compartments. In ecological networks, these compartments are sub‐networks with more links within compartments than between them. In microbial networks, specifically, modules and modularity are terms often used to refer to compartments and the degree of compartmentalization, respectively.

**Function:** The specific activity that a plant or microorganism performs, encompassing metabolic and biochemical processes that contribute to survival, growth, and interactions (plant–plant, plant–microbe, or microbe–microbe interactions).

**Functional capacity:** The functional potential of the microbial community typically determined by metagenomic assessment of the microbiomes' functional gene repertoire and/or molecular pathways. This is one way that scientists estimate the functional diversity of the microbial community.

**Functional diversity:** The variety of functions carried out by a microbial community. This term is often equated to functional richness, or the number of distinct functions present in a microbiome.

**Functional redundancy:** Multiple taxa within a community having one or more of the same or similar functional abilities (sometimes also referred to as functional overlap).

**Keystone microbe:** Microbial taxa that have a disproportionately large effect on their microbial community's diversity, structure, and/or function. Generally, these microbes are expected to have important functions that benefit the rest of the community, leading to increasing biodiversity. The keystone microbe concept is derived from the keystone species concept developed in community ecology of plants and animals, which identifies species that, despite making up a relatively small part of a community (in terms of biomass or abundance), have an important impact on their community, often increasing species diversity. Increases in diversity can arise from ecosystem engineering or other forms of facilitation. In microbiomes, highly connected hub taxa within microbial communities are more likely to be keystone microbes.

**Molecular mechanisms:** The genetic, epigenetic, and biochemical processes underlying a phenotype. In this paper, the genetic, epigenetic, and biochemical processes of interest are those underlying phenotypes that regulate plant–microbe interactions (e.g. the oscillations in nuclear calcium levels that create a cascade of transcriptional changes allowing for symbionts to colonize plants in arbuscular, ectomycorrhizal, and rhizobial symbioses).

**Plant–soil feedbacks (PSFs):** The response of plants to microbial communities derived from conspecific or heterospecific plants. PSFs are typically measured using plant biomass and can range from positive values (indicating that plants perform better with conspecific microbial communities) to negative values (indicating that plants perform worse with conspecific microbial communities).

**Stress legacy effects:** Changes in communities resulting from histories of stress that can affect how communities respond to their contemporary environment. Stress legacy effects can be transient (meaning community shifts following historical stress are temporary and transitional) or persistent (meaning the community shifts to alternative states following historical stress that persist even under different contemporary environmental conditions).

**Recovery:** The ability of a system to return to the original diversity, structure, or function/functional makeup after a perturbation.

**Resilience:** The ability of a system to withstand perturbation without fundamental changes to diversity, structure, or function. This includes both resistance to the perturbation and recovery from the perturbation.

**Resistance:** The ability of a system's diversity, structure, or function/functional makeup to remain unchanged immediately after a perturbation.

**Stable/stability:** Lacking fundamental changes to the diversity, structure, or function of a system through time and/or space (sometimes also referred to as invariability).

**Stress:** Environmental conditions that negatively impact an organism's survival, growth, and/or fecundity. While the stress often refers to abiotic environmental conditions (e.g. high temperatures, salinity, and drought), stress is also sometimes used to refer to biotic conditions that negatively impact organisms' survival, growth, or fecundity (e.g. competition). Stressful conditions are often inferred based on general knowledge (e.g. drought typically decreases plant survival and growth, making it a stressor), but individual species that are adapted to those conditions may not experience negative effects (e.g. cacti may be relatively unaffected by prolonged drought). When evaluating inter‐microbial and plant‐microbial dynamics under climate change, it is especially important to keep in mind that there is limited knowledge of the fitness consequences of presumed stressors for many microbial species.

Because climate change influences microbial community composition directly by selecting for tolerant or resistant microbial taxa, and indirectly through changes in plant–microbe interactions and plant community composition, it is difficult to disentangle whether microbes are primarily drivers or passengers of plant community responses to climate change. A rainfall manipulation in an Australian grassland found stronger support for climate‐mediated shifts in AM fungi driving plant community changes than vice versa (Deveautour *et al*., 2020). Further, changes in microbial abundance and community structure in response to drought can have long‐term effects on plant performance, even in the absence of later drought (Boyle *et al*., 2024), providing further evidence for the driver hypothesis. If microbes are usually drivers, then understanding microbial responses to stress will be key to predicting how plant communities are influenced by climate change.

### 2. Increasing salinity from sea level rise

The average sea level has risen by 111 mm over the past 30 yr, and this rate of sea level rise is expected to accelerate due to both thermal expansion of warmed seawater and melting of ice sheets and glaciers (Calvin *et al*., 2023), increasing salinity stress for organisms globally. Salinity is a complex stress that affects organisms both directly through ion stress from cytoplasmic accumulation of ions toxic to cells and indirectly through osmotic stress from high salt concentrations in soils, inhibiting water and nutrition acquisition (Liu *et al*., 2022). Many fungi and bacteria help plants tolerate salinity stress. For example, leaf endophytes can increase salt tolerance in native plants, and inoculation with rhizosphere microbes can enhance crop success under salinity stress (Rodriguez *et al*., 2008; Soares *et al*., 2016; Lei *et al*., 2025). Some microbes are more salt‐tolerant than plants and possess functional repertoires that can help hosts mitigate salt stress. Microbes alleviate salinity stress for plants by, for example, restoring ion balance (through decreasing Na^+^ uptake and compartmentalizing Na^+^ into vacuoles), restoring osmotic balance (through producing or stimulating plant production of osmolytes), increasing water use efficiency, or preventing plant cell damage by reducing the generation of reactive oxygen species (ROS) and stimulating plant ROS detoxification systems (Liu *et al*., 2022). Microbes can even augment the intrinsic salt tolerance adaptations of plants. For instance, red mangroves – an important coastal tree species – have mechanisms to cope with salinity such as root ultrafiltration and salt sequestration (Tomlinson, 2016). However, red mangroves inoculated with endophytes that have experienced salinity stress grow better under high salinity than uninoculated trees or those grown with endophytes without a salinity history (Subedi *et al*., 2022). Microbially mediated salinity tolerance in plants may have broader impacts on ecosystem resilience (Box 1) to climate change. For example, mangroves are important foundation species that reduce coastal erosion from hurricane storm surge (Pennings *et al*., 2021), and in some coastal systems, plant‐driven accumulation of organic matter contributes to vertical land development, enabling coastal ecosystems to keep pace with sea level rise (Cahoon *et al*., 2021).

Salinity also affects microbial community diversity and structure. Several studies found fungi can better tolerate osmotic stress from salinity than bacteria, suggesting fungal chitinous cell walls provide salinity protection (Haj‐Amor *et al*., 2022). However, other studies have found bacteria to be more tolerant, emphasizing the need to understand mechanisms underlying *how* microbes respond to salinity changes to develop a more predictive framework. Interestingly, salinity can decrease microbial extracellular enzyme activity, which is important in both microbial acquisition of nutrients and processes that affect plants, like decomposition and nutrient cycling. Salinity can disrupt enzyme activity by not only reducing microbial biomass in soils but also by directly affecting enzyme structure (Haj‐Amor *et al*., 2022). Salinity‐driven shifts in plant root exudates are common and can be used as a cry‐for‐help signal to recruit stress‐reducing bacteria (Kumar *et al*., 2023). However, salinity may also disrupt the establishment of some important plant‐microbial mutualisms through effects on soil signaling. For instance, while AM fungi can ameliorate salinity effects on host plants (Boorboori & Lackóová, 2025), salinity can reduce the production of strigolactones (López‐Ráez, 2016), a diffusible signal important for host detection of AM fungi. Salinity stress from climate change may thus disrupt the establishment of microbe–plant interactions, even when the resulting interactions would be beneficial.

### 3. Changing winter timing, snowpack, and snowmelt

Winters are warming and drying, and late spring frost events are increasing, with major consequences for snow cover, microbial‐mediated processes, and plant phenology and productivity (e.g. Contosta *et al*., 2024; Gottlieb & Mankin, 2024). While plants are largely dormant during winter, microbial activity persists beneath snowpack, leading to a ‘vernal flush’ of nutrients available with snowmelt (e.g. Sorensen *et al*., 2020). Early snowmelt can extend or shorten the growing season for plants and microbes (Contosta *et al*., 2024). Early emergence of plants, due to warming air temperatures, can make their leaves more susceptible to frost damage (Bigler & Bugmann, 2018). Plant endophytes, both fungal and bacterial, can benefit plants under cold temperatures by increasing acclimation via hormonal signaling and increasing osmolyte concentrations to ward off freezing, but few studies have explored whether microbes benefit plants during winter stress in natural ecosystems. While aboveground plant growth often advances with earlier snowmelt and can be subjected to freezing events, root phenology can be less responsive (e.g. Blume‐Werry *et al*., 2017). However, experimental increases in snow depth have caused either increases (Li *et al*., 2020) or decreases (D'Imperio *et al*., 2018) in root production, highlighting the context dependency of belowground plant responses, which may influence belowground microbial responses. Diversifying partner mutualists or associating with mutualists with particularly valuable functional traits may provide greater plant resilience to changing winters. For instance, tree species forming dual mycorrhizal associations show greater resilience to cold and drought, with drought tolerance associated with higher frequencies of AM fungi and obligate symbioses, and cold tolerance linked to ectomycorrhizal associations and facultative symbioses (Laanisto *et al*., 2025), suggesting that dual colonization may buffer trees against changing winters (Kivlin *et al*., 2013).

Changing winters also have multiple direct and indirect effects on microbial communities. Loss of insulating snow cover exposes soils to frost, damaging roots and thereby reducing resources for soil microbes (Comerford *et al*., 2013). Additionally, drier soils resulting from early snowmelt can favor deeper rooted plant species (Liu *et al*., 2018), shifting plant and microbe community traits and interactions. In harsh high‐elevation environments, in contrast to lowland grasslands, there is a notable increase in species associated with the Acaulosporaceae family, which have low metabolic costs and invest less biomass in extraradical hyphae and internal root structures (Chagnon *et al*., 2013) – traits that could give them an advantage in drier, early snowmelt conditions.

Changes in snow properties (e.g. depth, density, and duration) regulate albedo light reflection, soil temperatures, spring meltwater availability, and nutrient cycling (Contosta *et al*., 2024), which can trigger seasonal transitions in the phenologies of plants and microbes (Flynn & Wolkovich, 2018). Plant‐associated microbes can respond to changing snowmelt along with their hosts or independently (Millar & Bennett, 2016). Microbial activity and phenology are cued by nutrient flushes, moisture, and temperature (Ruehr & Buchmann, 2010; Yin *et al*., 2022), while plant phenology primarily follows temperature and photoperiod (Flynn & Wolkovich, 2018), suggesting plant and microbial communities may be decoupled under altered winter conditions. In mountain meadows, for example, different microbial groups dominate under snow, during snowmelt, and post‐melt, with microbial biomass crashes and nitrate flushes upon snowmelt (Sorensen *et al*., 2020). In these ecosystems, mycorrhizal fungi increase in abundance during spring, suggesting that coupled plant‐mycorrhizal phenology may help conserve nutrients, a dynamic that could be disrupted by shifts in snowmelt timing (Sorensen *et al*., 2020). Our understanding of the ecological impacts of decoupled interactions could benefit from more research that explicitly tests how decoupling influences ecosystem functioning and whether decoupled interactions can recover, either through partnerships with novel species or through adaptation to changing climate conditions. To advance this goal, we advocate not only for long‐term assessments linking ecosystem functions to the degree of phenological mismatches but also for experimental manipulations that disrupt coupled interactions and/or introduce novel partners with overlapping phenological windows.

### 4. Intensification of extreme events: hurricanes, flash floods, and fires

Climate change has increased the frequency and severity of extreme events that have immediate and strong impacts on ecosystems. These include stronger hurricanes, increased flash floods, and more frequent wildfires (Calvin *et al*., 2023). Our current knowledge of the effects of extreme events on plant–microbe interactions depends on the event type. For instance, hurricanes are difficult to study because they are unpredictable and cannot be readily experimentally manipulated, leaving their effects on plant–microbe interactions understudied. By contrast, the long history of fire ecology and the use of prescribed burns for management have fueled extensive knowledge and toolkits to study the increased frequency and intensity of fires. By imposing strong abiotic filters on communities, extreme events can meaningfully impact plant and microbial diversity and their interactions.

#### Hurricanes

As global temperatures rise, warmer ocean surface waters are increasing both hurricane frequency and intensity (Calvin *et al*., 2023). Little is known about how microbes help plants withstand hurricanes. However, direct impacts of hurricanes on microbiome composition likely influence plant–microbe interactions. High‐intensity winds and flash flooding can redistribute soils, vegetation, and litter across landscapes, which may homogenize microbial communities by reducing variation in taxonomic and functional diversity across landscapes (DeLeon‐Rodriguez *et al*., 2013). Hurricanes have been shown to restructure the composition and function of aquatic microbiomes (Amaral‐Zettler *et al*., 2008; Walker *et al*., 2022). For instance, decreased presence of important functional gene pathways for carbon cycling and primary production in marine microbiomes after Hurricanes Matthew (2016) and Florence (2018) suggests that hurricanes can be disruptive for microbiome function (Garrison *et al*., 2022). Less is known about hurricane effects on terrestrial microbiomes. However, one study found that Acidobacteria increased rapidly in Costa Rican forest soils during the first 2 years after Hurricane Otto (2016), followed by a shift toward fungal decomposers that likely targeted remaining, slow‐to‐decompose canopy debris in subsequent years (Eaton & McGee, 2025). Evaluating hurricane‐driven effects on the functional capacity (Box 1) of soil microbiomes using metagenomics and metatranscriptomics would be valuable for increased mechanistic understanding of hurricane effects on decomposition and nutrient cycling (factors that could affect the rate of plant community recovery). Of particular interest is whether hurricanes disrupt microbial mutualisms that are important in plant recovery post‐hurricane. In addition, rhizosphere and phyllosphere microbial mutualisms likely impact host plant resistance to hurricane‐driven stressors. For instance, research has shown that microbes can influence root architecture and investment (Kannadan & Rudgers, 2008; Malicka *et al*., 2022), both of which can be important in determining whether plants withstand hurricane‐force winds. The next steps in developing our understanding of hurricane effects on microbiomes include pre‐ and post‐hurricane microbiome assessments to determine the degree to which hurricanes cause taxonomic and functional biotic homogenization of soil microbiomes, which landscape features promote resistance and/or recovery of microbial communities after hurricanes, and the consequences of these microbial changes for plant communities and ecosystems.

#### Floods

Flood risk has steadily increased across all continents since the 1950s and is expected to increase globally due to climate change. Climate change creates warmer air that can hold more water, facilitating both sustained and flash flooding (Calvin *et al*., 2023). Flooding creates anoxic soil conditions that harm or kill terrestrial plants through accumulation of toxic substances, carbon starvation, cytoplasmic acidification, and/or disease (Martínez‐Arias *et al*., 2022). Plant growth‐promoting bacteria from the genera *Bacillus*, *Microbacterium*, *Methylophaga*, and *Paenibacillus* can protect against flooding stress by reducing ethylene accumulation, counteracting the detrimental effects of high ethylene levels on plant development. Other bacteria like *Pseudomonas putida* can counteract the inhibitory effects of hypoxic stresses on plant biomass (Martínez‐Arias *et al*., 2022). Some soil anaerobic microbes appear to induce flood tolerance by secreting organic acids that cause the development of ‘radical oxygen loss’ barriers (Colmer *et al*., 2019). Flooded microbial communities shift in activity and structure in response to these same abiotic stressors that plants face (Graff & Conrad, 2005; Wagner *et al*., 2015; Knight *et al*., 2024). Microbial resistance to flooding is phylogenetically conserved, leading to predicted shifts in functional and taxonomic diversity with increased flooding (Knight *et al*., 2024). Furthermore, spatiotemporal variation in flood duration and frequency influences microbial community responses, including altering the relative abundance of plant pathogens and mutualists (Wagner *et al*., 2015; Francioli *et al*., 2022). Despite these findings, few studies have linked these changes to interactions with plants or effects on plant communities.

#### Fire

Increased aridity and fuel loads from fire suppression are leading to intense and difficult‐to‐control fires (Calvin *et al*., 2023), and both contemporary and past fires play a crucial role in how microbes affect plant performance. For instance, Revillini *et al*. (2022) found that recent fires have a greater influence on how microbes affect native plant germination and growth than more distant fire history. Yet, microbiomes with fire histories consistent with fire intensification showed greater downregulation of nutrient cycling genes in response to new fires (Revillini *et al*., 2025), indicating that past fire can also shape current community function. Heat, changes in soil pH, and increased soil albedo act as abiotic filters that can reduce microbial biomass and activity (Hu *et al*., 2023; Ibáñez *et al*., 2025) and favor heat‐tolerant taxa (Johnson *et al*., 2023). Lost microbial diversity following fire may be responsible for the homogenization of microbial effects across plant species (Senior *et al*., 2018; Warneke *et al*., 2023), which can reduce the importance of plant–microbe interactions in structuring plant communities. Fungal communities are more sensitive to fire and have a slower post‐fire recovery than bacterial communities (Fultz *et al*., 2016; Pulido‐Chavez *et al*., 2023). However, variation in fire intensity can alter the composition and/or the recovery time of microbial communities (Dove *et al*., 2021; Hu *et al*., 2023), adding additional complexity to potential fire effects. Fire also alters microbial beta‐diversity by freeing niche space, increasing the importance of stochastic processes such as dispersal (Ferrenberg *et al*., 2013; Dove *et al*., 2021), though deterministic processes can regain dominance over time (Ferrenberg *et al*., 2013). This alteration of assembly mechanisms and microbial dispersal via smoke (Ellington *et al*., 2024) could lead to lower beta‐diversity between comparable habitats, complicating predictions for regional plant diversity.

## III. Burgeoning advancements in climate change effects on beneficial plant‐microbial interactions

Given how crucial plants and microbiomes are for ecosystem function and the multiple ways climate change affects these organisms, we must develop a predictive framework for when beneficial plant‐microbial interactions will continue supporting plant productivity, plant diversity, and microbial services. In this section, we discuss conceptual areas that inform important gaps identified in our review of climate change stressors in the previous section. Specifically, we discuss the need to (1) understand how climate variability influences plant–microbe interactions, (2) determine what attributes make a microbial community resilient to climate stressors, (3) evaluate how abiotic and biotic legacies interact with contemporary climate change to affect microbiomes and plant‐microbial interactions, and (4) identify the molecular mechanisms underlying how microbiomes help plants tolerate and/or resist climate change. These research areas, while not exhaustive, spotlight efforts that show promise for improving our abilities to predict how climate change reshapes plant–microbe interactions and the ecosystem services they provide and to find strategies to potentially mitigate their disruption.

### 1. Climate variability: Potential for interactive effects among changing climate parameters

**Fig. 2:**
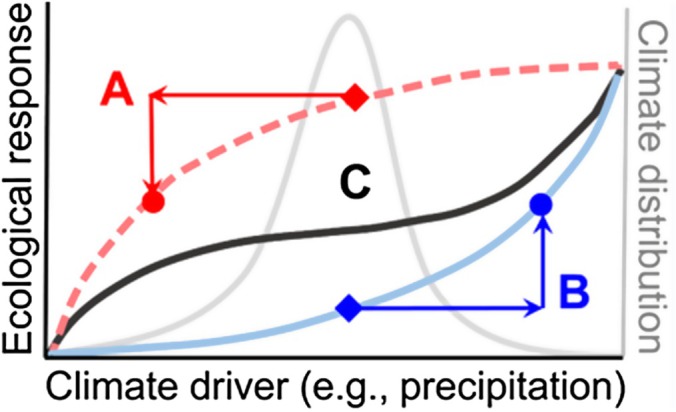
Evidence for interactions between mean and variance in climate change. According to Jensen's inequality, a concave nonlinearity (A, red function) predicts a cost of variability because the ecological response declines strongly at one or both climate extremes (here, extreme dry). By contrast, a convex nonlinearity (B, blue function) predicts that more variability will be beneficial because the ecological response increases strongly under at least one climate extreme (here, extreme wet). A complex climate sensitivity function (C, black function) indicates that the net costs or benefits of increasing climate variance depend on the mean climate, the signature of a mean × variance interaction. The gray curve represents the climate distribution (here, precipitation).

Climate forecasts project change in two key parameters of the distribution of climate variables: the mean (e.g. gradual warming) and the variance (e.g. drought‐deluge dynamics, Fig. 2, gray climate distribution), and these dual changes could interactively influence plant–microbe interactions. Although changes in mean temperature and precipitation are expected globally, most regions will also receive more variable future precipitation regimes (Calvin *et al*., 2023). For example, the climate in the Western United States has become both more arid and more interannually variable during the past 100 years (Maurer *et al*., 2020; Harp & Horton, 2023). Although forecasts predict that dual changes in climate mean and variance will continue to intensify, few studies manipulate these changes jointly to anticipate their combined effects (but see Benedetti‐Cecchi *et al*., 2006; Burgmer & Hillebrand, 2011). If variance in climate has strong effects on ecological responses (Fig. 2), current approaches will fail to predict ecological change because they largely ignore the influence of escalating variability in climate.

Beneficial plant–microbe interactions provide tractable and interesting systems to evaluate theory about the ecological importance of climate variability because microbes can respond much faster to environmental variability than plants. Theory predicts that temporal variability in climate can have powerful ecological and evolutionary consequences due to nonlinearities in biological responses to the environment (Jensen, 1906) and the stochastic nature of climate (Adler & Drake, 2008; Ridolfi *et al*., 2011). The effects of temporal variability in climate depend on the shape of a ‘climate sensitivity function’ that relates an ecological response to its climate driver (Fig. 2, Rudgers *et al*., 2018). Nonlinear sensitivities signal that climate variability is important *even if the mean climate does not change* (Jensen's inequality, Jensen, 1906). A convex nonlinearity (e.g. Fig. 2, blue function) means that more variability will be beneficial to the measured ecological response because the response increases strongly under at least one climate extreme (here, extreme wet). For example, global analysis of primary productivity revealed that greater variability in interannual precipitation was associated with higher productivity in arid ecosystems that had < 300 mm mean annual precipitation (Hou *et al*., 2021). By contrast, a concave nonlinearity (Fig. 2, red function) represents a net negative effect of variability because the ecological response declines strongly at one or both climate extremes. Similar benefits or costs of variability could accrue to plant–microbe symbioses, but perhaps more importantly, symbioses could alter the shape of the climate sensitivity function for plants. Finally, a complex nonlinearity (Fig. 2, black function) indicates a mean × variance interaction. Here, the shape of the nonlinearity flips from net benefits of variability (convex) under a wet climate mean to net costs (concave) when it is dry. Nonlinearities predict instability in ecological responses under large temporal variability in climate. By contrast, linear relationships signal sensitivity in ecological responses to change *only* in mean climate. It remains unresolved how commonly plant–microbe symbioses have nonlinear relationships with climate variables or the degree to which the symbiosis alters sensitivity. Analysis of long‐term data using this approach predicts that rising temperatures could alter the nonlinearities that determine the costs and benefits of variance in precipitation for net primary production in several dryland ecosystems, which has significant consequences for biodiversity and ecosystem services (Rudgers *et al*., 2018).

Climate sensitivity theory has promise for predicting future dynamics of the highly variable, context‐dependent interactions between plants and microbes. Long‐term observations can detect nonlinearities that signal the importance of climate variance, or reveal signatures of mean × variance interactions (Sasaki *et al*., 2023). However, such analyses have not been applied to microbes because long time series collected over naturally temporally variable climates are exceedingly rare. Thus, despite traction in population ecology (Lawson *et al*., 2015), few observational studies of multiple species have tested for complex nonlinearities (but see Kazenel *et al*., 2024), and none, to our knowledge, have explored such dynamics for plant–microbe interactions. Moreover, experiments are required to detect causality for the impacts of increasing climate variance (Rudgers *et al*., 2023). In a unique field experiment that amplified variability in precipitation, soil fungi had generally low sensitivity, but fungal diversity declined, possibly due to declines in host plants caused by increased precipitation variability (Louw *et al*., 2022). This result suggests that considering plant–microbe interactions may be important for accurately predicting how climate variance affects ecosystem structure and function, which is critical to conservation prioritization and effective management.

### 2. Stability of microbial biodiversity and function

**Fig. 3:**
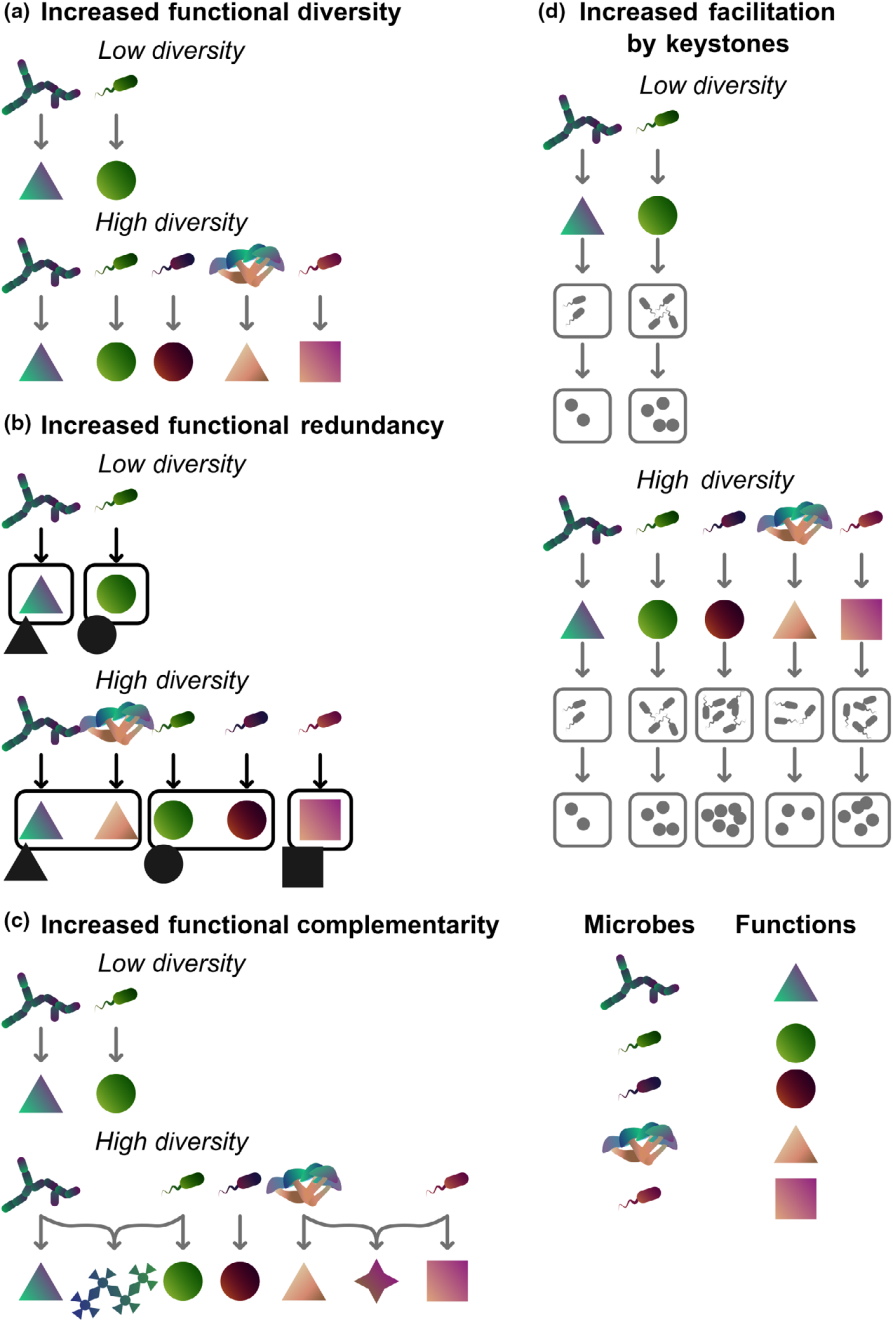
Ecological theory predicts that taxonomic diversity in communities can lead to greater functionality due to increased functional diversity (a), increased functional redundancy (b), increased functional/metabolic complementarity (c), and increased facilitation by keystone microbes (d). In (a), if a community has more microbial taxa that also have some unique functional capabilities, high taxonomic diversity will result in a greater diversity of functions (i.e. greater functional diversity). In (b), if a community has many microbial taxa, there are more chances that each function will be replicated in more than one microbial taxon (functional redundancy). If a taxon is lost due to stress or perturbation, the functions it possessed can still be retained within other members of the community. In (c), if a community has more microbial taxa with different functions or metabolic repertoires, there are more opportunities for complementarity of functions provided by different microbes or metabolic complementarity that can lead to the production of other important functions or resources (e.g. multiple microbes contributing enzymes needed to break down cellulose) (functional/metabolic complementarity). In (d), if a community has many microbial taxa, there are more chances for it to include taxa with functional attributes that facilitate the persistence of other microbial taxa, which themselves provide different functions (facilitation). Microbes that facilitate many taxa by producing important functions can be ‘keystone microbes’. Facilitated microbial taxa are represented in this panel as small gray microbes within gray boxes, and the functions provided by these facilitated taxa are represented as small gray circles within the gray boxes.

Building a better predictive framework to identify which plant microbiomes will be most stable and will consistently provide functional benefits under climate change requires a comprehensive understanding of resilience – that is the ability to withstand perturbation without fundamental changes to structure (diversity, composition, architecture) or function (Box 1). Traditionally, ‘stable and healthy’ microbiomes are those that have high alpha diversity and low species turnover (Williams *et al*., 2024). Ecological theory and empirical studies have linked taxonomic diversity to promoting function within communities through multiple mechanisms (see Fig. 3 for a discussion of these mechanisms). Often, high taxonomic diversity stabilizes microbiomes because a larger species pool increases opportunities for both functional diversity and functional redundancy (Box 1; Fig. 3; Wagg *et al*., 2019). However, not all taxonomically diverse microbiomes automatically have high levels of functional redundancy (Delgado‐Baquerizo *et al*., 2016), and communities with low species turnover may actually be shifting significantly in terms of function (Williams *et al*., 2024). Thus, to accurately assess a microbiome's stability, additional information beyond taxonomic diversity and turnover is needed, such as the microbial communities' network structure and functional attributes.

**Fig. 4:**
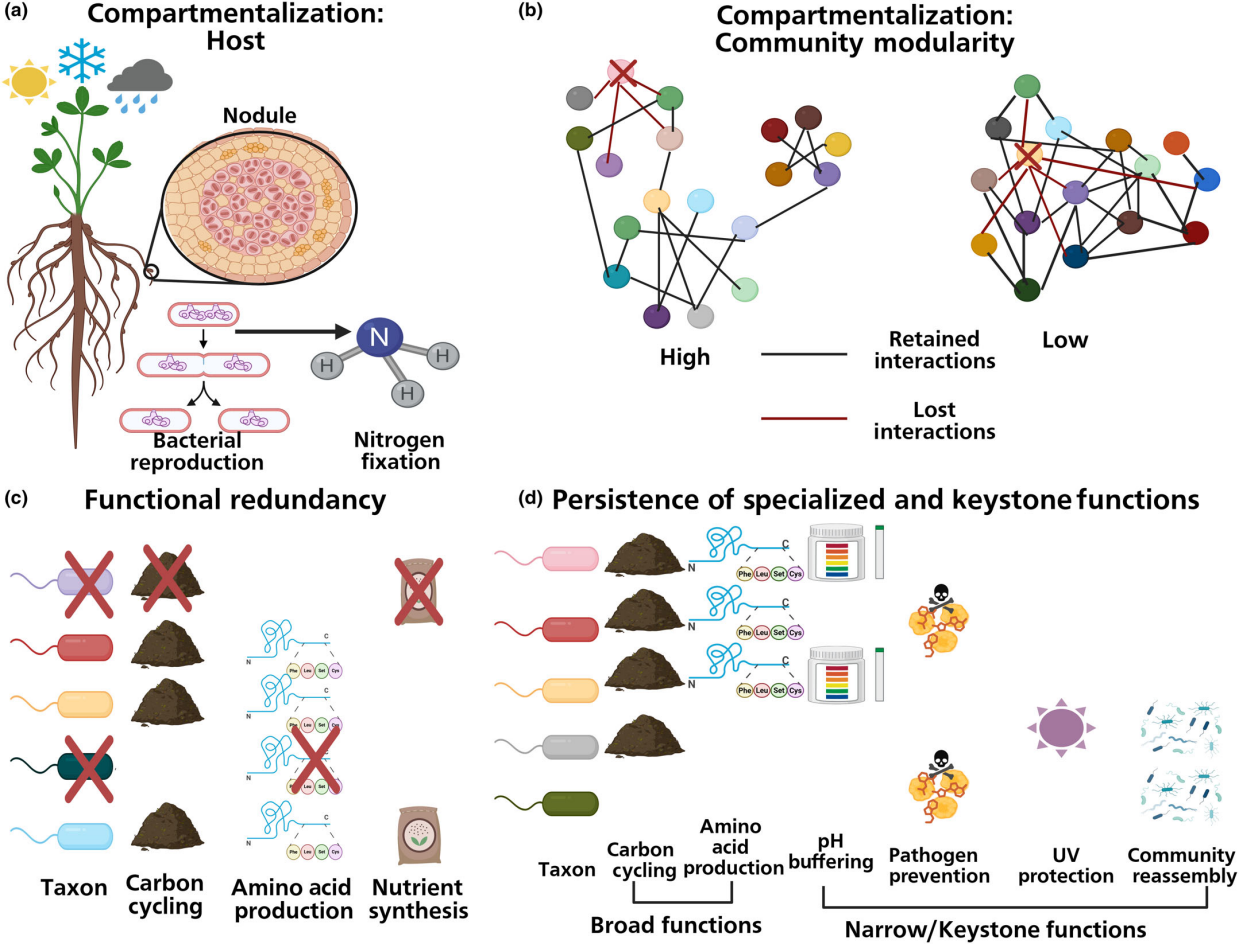
Mechanisms of resilience. (a) Host compartmentalization spatially separates mutualists across plant organs, which allows host plants to have fine‐tuned control over symbioses to maximize microbial rewards across a diversity of abiotic conditions. (b) Community compartmentalization into distinct modules (i.e. high modularity) promotes stability since if a taxon is lost, only the module containing that taxon is impacted rather than the community at large. (c) If microbe species go locally extinct but there is high functional overlap among taxa (i.e. functional redundancy), functions are retained within the ecosystem. Functional redundancy is most common in broad functions such as carbon cycling, amino acid production, and nutrient synthesis. (d) Narrow functions often have less functional overlap than broad functions and include important keystone functions such as buffering pH, pathogen prevention via reduced establishment of invasive microbes, protection from UV and other harsh conditions, and driving community reassembly after perturbations. These critical functions must persist in order for a community to recover after a perturbation (created in BioRender. Afkhami, M. (2025) https://BioRender.com/zev00pj).

The main ecological mechanism proposed for the resilience of microbiome structure is compartmentalization. Compartmentalization (Box 1) is the division of communities into distinct groups or compartments where taxa within a compartment have stronger associations with each other than with taxa in other compartments. Compartmentalization includes both mutualists that spatially separate across plant organs and distinct modules of co‐occurring/interacting free‐living microbes in the environment (e.g. soil microbiomes). By spatially compartmentalizing mutualists (e.g. legumes housing rhizobia bacteria in nodules), plants can have fine‐tuned control over symbionts and increase microbial rewards (e.g. shift rhizobia energetic investment away from reproduction toward nitrogen fixation), enabling these benefits to be retained across a variety of environmental conditions (Fig. 4a; Chomicki *et al*., 2020). Formation of distinct modules (i.e. interconnected groups) within environmental microbiomes can compartmentalize interspecific interactions within diverse free‐living microbial communities. If one taxon is lost due to a perturbation, effects are predominately limited to the module containing that taxon, while the other modules are relatively unaffected (Kajihara & Hynson, 2024) (Fig. 4b). Modularity has recently predicted the stability of soil communities in a natural grassland ecosystem (Zhang *et al*., 2024) and in agriculture (Xiao *et al*., 2024).

Additional mechanisms of microbial functional resilience include functional redundancy, persistence of specialized functions, and maintenance of microbe–microbe interactions. Functional redundancy is when the function associated with the loss of one taxon is fulfilled by one or more other taxa with a similar function within host‐associated or free‐living microbiomes (Allison & Martiny, 2008; Box 1; Fig. 4c). There has been an upsurge of studies indicating microbial functional genes are strongly tied to environmental niche partitioning and that many diverse taxonomies can fulfill equivalent functional roles for both the microbiome and plant host (Louca *et al*., 2018; Barnes *et al*., 2020; Zeng *et al*., 2023; Ramond *et al*., 2025). A recent meta‐analysis of environmental microbiomes spanning many diverse freshwater, marine, and terrestrial ecosystems supports functional redundancy as a common property of numerous host‐associated and free‐living microbiomes globally (Puente‐Sánchez *et al*., 2024). Functional redundancy is also significantly more common in ‘broad’ functions like carbon cycling and amino acid production that many microbes can perform compared with ‘narrow’ functions like nitrogen, phosphorus, and potassium metabolism, which can be performed by relatively few keystone microbes (Box 1) (Chen *et al*., 2022). This indicates that even communities with high overall redundancy are susceptible to the loss of specialized functions. Consequently, the persistence of keystone microbes stimulates resilience since these microbes are able to shelter the host/community from environmental extremes like UV radiation (Zhang *et al*., 2025), modulate the severity of abiotic shifts such as pH (Xun *et al*., 2021), prevent the establishment of invasive competitors (Hu *et al*., 2020), and drive community reassembly after major perturbations (Rawstern *et al*., 2025) (Fig. 4d). Additionally, a microbe's contribution to overall community function (i.e. suite of genes transcribed) and impact on microbe–microbe interactions can shift in different environments (Fetzer *et al*., 2015; Spratt & Lane, 2022). In sum, microbes that retain their functions, especially rare keystone functions, across many environmental conditions can enable stable inter‐microbial interactions and promote resilience.

Another ecological mechanism that has been considered to promote stability within the microbiome is the limitation of positive microbe–microbe interactions (Coyte *et al*., 2015), because cooperation begets interdependency of microbes, creating the potential for simultaneous species loss. For instance, when microbes rely on other microbes for their growth and survival (e.g. cross‐feeding on a metabolite produced by another microbe), loss of the microbe producing the nutrient can create a positive feedback loop in which the other microbes relying upon that nutrient are also lost, and the microbes relying upon those microbes are next lost, and so on, potentially destabilizing the whole microbial community. This destabilization effect has been documented in plants with microbiomes that have strong interspecific facilitative microbe–microbe interactions and weakened negative microbe–microbe interactions since they were more susceptible to destabilizing effects of pathogen overgrowth and disease (Li *et al*., 2019; Wang *et al*., 2024). While the dampening of positive interactions may promote microbial community stability, paradoxically it may also destabilize the microbiome's coexistence with the host plant, where the host may no longer invest as many resources to maintain the relationship. Plant‐associated microbiomes that have intense negative inter‐microbial interactions can be detrimental to the host since competition among microbes both generates pressure to exploit the host and diverts microbial resources away from the host (Afkhami *et al*., 2020). By contrast, cooperating microbes within microbial communities can synergistically improve host performance and available rewards, which may promote the longevity of the host–microbe relationship (Afkhami *et al*., 2020, 2021). Evidence in a variety of plant systems suggests that host‐associated microbiomes have either higher (soybean rhizosphere: Liu *et al*., 2019) or lower (duckweed: Laurich *et al*., 2024) incidences of positive interactions than free‐living microbiomes. Therefore, whether negative or positive microbial interactions stabilize plant microbiomes likely depends upon environmental context and whether microbial interactions promote benefits for all or maintain microbial diversity. In mild stress, the costs of cooperation are high, and strong negative feedbacks, where microbes limit one another's abundances, can promote coexistence among microbes to maintain a diverse community that supports functional redundancy (Oliveira *et al*., 2014; Solowiej‐Wedderburn *et al*., 2025). As stress becomes more severe, the benefits of cooperation may outweigh the costs, and strong positive feedbacks can help speed recovery because repeatedly positive interactions have accelerating benefits for all parties (Cavaliere *et al*., 2017; Amarnath *et al*., 2023).

To encompass the complexities of host–microbe, microbe–microbe, and microbe‐environmental interactions under climate change, future modeling frameworks for microbiome stability should not only incorporate the traditional metrics of diversity and taxonomic turnover, but also include community modularity, functional redundancy, predicted persistence of keystone taxa and functions, and maintenance of microbial interactions. Much of our current theory on host–microbiome stability stems from human gut microbiome research (Lozupone *et al*., 2012; Coyte *et al*., 2015; Chen *et al*., 2021). To build more comprehensive models applicable to plant systems, more experiments with direct perturbations of plant microbiomes in nature are needed to decipher which attributes of plant‐associated microbiomes best predict response to climate change. For example, microbial networks could be used as tools to calculate modularity and microbe–microbe interactions, while shotgun metagenomics could capture functional data for a variety of plant‐associated microbiomes at baseline conditions. Then a perturbation, such as drought, could be applied and tested to determine which of these properties best predicted whether a community was resilient or divergent.

### 3. Stress legacies of microbiomes: How past stress influences future resilience

Stress legacies (Box 1) of microbiomes – changes in microbial communities resulting from their prior exposure to stress – can impact how plant–microbiome interactions respond to contemporary climate‐related stress, thereby influencing plant tolerance to climate change. Drought, heat, and salinity legacies can improve a microbiome's ability to enhance plant performance under the same stressor in the future (Lau & Lennon, 2012; Xu *et al*., 2018; Subedi *et al*., 2022; Allsup *et al*., 2023). Over time, these effects could impact plant evolution by buffering plants against negative fitness effects of contemporary stress (Bolin & Lau, 2024). Thus, applying stress during cultivation of microbial inocula or sourcing inocula from stressful environments could provide a novel strategy for enhancing plant resilience to climate change (Afkhami, 2023; Allsup *et al*., 2023).

**Fig. 5:**
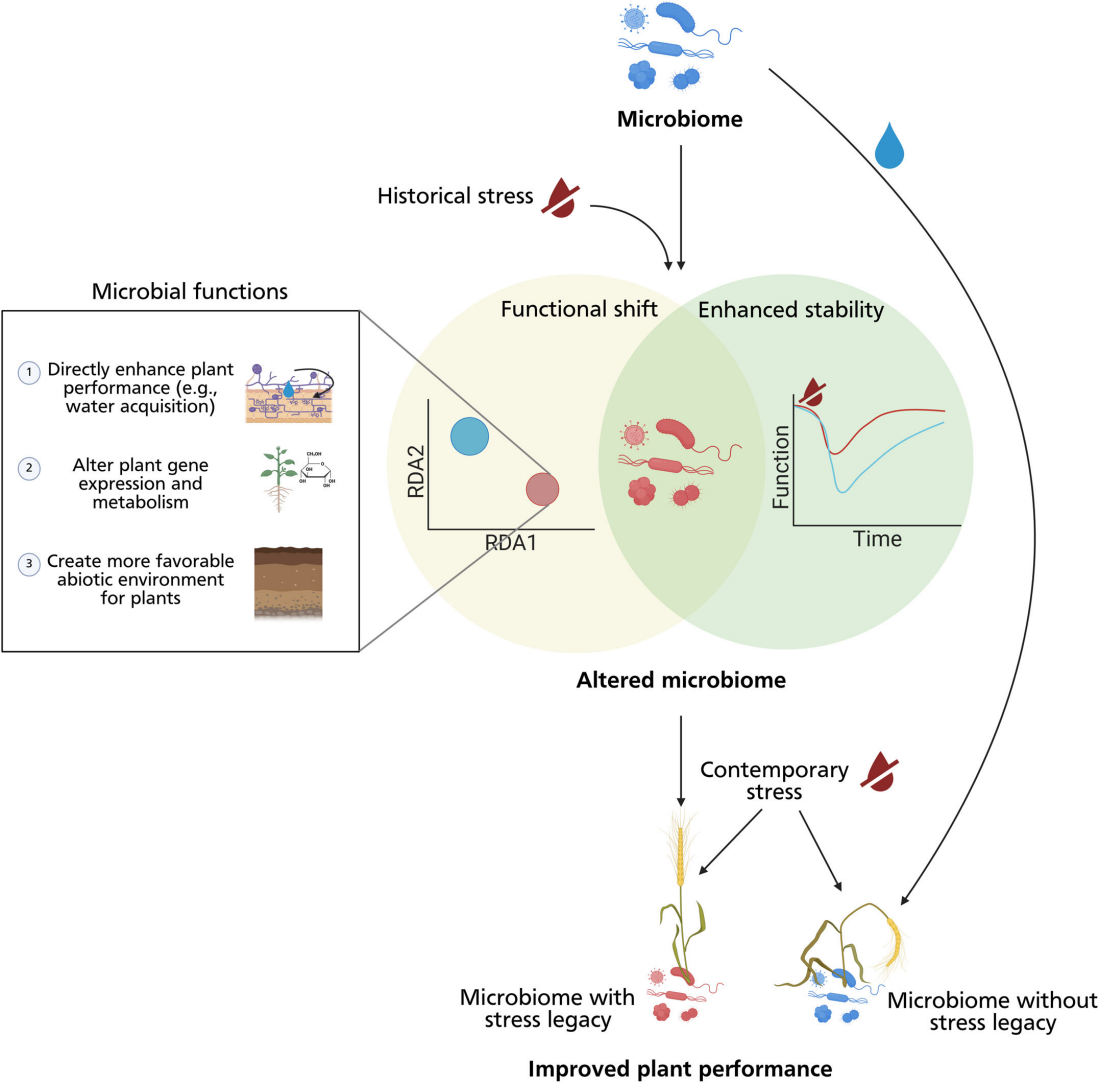
Framework for proposed pathways through which legacies of climate stress alter microbial ability to ameliorate contemporary stress for plants. Experience with historical climatic stressors, which are exhibited as stress legacies, can alter microbial function and/or enhance microbiome stability under contemporary stress, allowing for improved plant–microbiome interactions under contemporary stress. Struck‐through red water droplets represent a climate stressor (e.g. drought), blue microbes represent microbiomes without past experience with the climatic stress, and red microbes represent microbes with a stress legacy resulting from past experience with the climatic stress (created in BioRender. Afkhami, M. (2025) https://BioRender.com/k6krhef).

We still lack a clear understanding of *how* historical stress creates legacies that underlie beneficial outcomes under contemporary stress, including what shifts in microbial communities follow historical stress, the role of plants in shaping legacy effects, and how different properties of legacy stressors lead to differential plant‐microbial interaction outcomes. Broadly, microbial stress legacy effects may operate through two nonmutually exclusive pathways: functional changes in microbiomes or changes in microbiome resilience (Fig. 5). In both pathways, prior stress exposure can shape microbiomes in ways that either produce persistent legacy effects, through the establishment of alternative microbial stable states (Hawkes & Keitt, 2015), or transient legacy effects, via time lags or priority effects, which could also lead to persistent legacy effects in the longer term (Vass & Langenheder, 2017). In the first pathway, historical stress selects for microbiomes that can benefit their host, such as by directly enhancing plant growth or alleviating abiotic stress directly. Mechanisms could include species sorting, evolution within species, and horizontal gene transfer. In the second pathway, prior exposure to stress selects for resilient microbiomes, thus stabilizing microbiome functions that are beneficial to plants under contemporary stress conditions (Bardgett & Caruso, 2020).

The principal challenge arising from this framework is pinpointing exactly what altered microbiome properties drive effects on plant performance. Compositional shifts resulting from historical stress are well documented (Lau & Lennon, 2012; Xu *et al*., 2018; Allsup *et al*., 2023) and may be important for plant performance. For example, prior exposure to drought can enhance both the microbiome compositional and functional resistance to contemporary drought, suggesting a link between compositional and functional responses (Ochoa‐Hueso *et al*., 2018). However, functional changes do not always mirror compositional changes (discussed in section: Stability of Microbial Biodiversity and Function). It is also unclear whether legacy effects act through direct selection on communities and their functions or via changes in microbiome resilience (Fig. 5), and which microbial functions are most affected, such as those that help plants access resources, rewire plant gene expression and metabolic activity, or alter abiotic environments. Some studies show that stress legacies select for microbes that lead to functional shifts in microbiomes as well as shifts in plant and microbial metabolism. For instance, when used as inoculum, isolates from drought‐selected microbiomes led to enhanced plant growth, shifts in root microbial composition, and altered plant and microbial metabolism under drought (Xu *et al*., 2018). In another system, microbes from salt‐exposed rhizospheres improved plant performance under salinity and microbiome resistance to salinity, and were enriched for genes related to salt stress acclimatization, nutrient solubilization, and competitive root colonization (Bharti *et al*., 2014, 2015; Yuan *et al*., 2016). These findings suggest historical stress creates legacies by selecting for traits that benefit both microbes and host plants under contemporary stress (Fig. 5).

Importantly, plants may play an active role in producing legacy effects, both through altering how microbes respond to historical stress and through how they themselves interact with microbiomes with stress legacies. This emphasizes the need to determine whether legacies are plant‐mediated or microbial‐driven. The existing literature on plant priming has shown that plants can generate root exudates that filter their microbial communities (Seitz *et al*., 2022; Zhang *et al*., 2023), and the host control literature has shown that in some cases plants can allocate resources to preferred partners, encouraging stable and beneficial interactions (Bever *et al*., 2009; Wilde *et al*., 2024). Thus, plants may mediate the effects of microbial stress legacies through selecting microbial taxa that are beneficial under contemporary stress, and many growth‐promoting and stress‐ameliorating microbes are isolated from plants from stressful environments (Gehring *et al*., 2017; Kozaeva *et al*., 2024) (see Plant–Soil Feedbacks section). For instance, drought‐exposed plants were able to select for microbes that enhanced plant performance under drought when used as inoculum, likely through altering the expression of metabolic genes and thus root metabolite concentrations (Xu *et al*., 2018). Furthermore, different plant species may respond differently to microbiomes with stress legacies (Meisner *et al*., 2013; Revillini *et al*., 2022). Taken together, these suggest that plants influence how historical stress produces stress legacy effects on microbes as well as how legacy effects in microbiomes translate into impacts on plants, but how plants achieve this and how this varies among plant species require further investigation. Disentangling the extent to which stress legacies in microbiomes are plant‐driven vs originate from the microbiome is central to understanding their mechanisms.

To improve predictions as to when stress legacies will be important for plants and ecosystem functions, we also need to understand what attributes of historical stress (e.g. its duration, intensity, and type) enhance or decrease the strength and persistence of their effects. For instance, microbial impacts on plant performance may be more strongly shaped by recent and not historical fire (Revillini *et al*., 2022), whereas long‐term precipitation alters the sensitivity of resource acquisition gene abundance to current soil moisture (Broderick *et al*., 2025). This suggests historical context could be important for modifying microbiome function under contemporary conditions for some stressors but not others, but the underlying reasons are unclear. Potentially, long‐term stressors such as climate could be more effective at driving microbial adaptation than short‐term pulse stressors that may allow for subsequent recovery. Alternatively, extreme contemporary stressors such as fire could push microbiomes to alternative stable states in ways that outweigh or are blind to historical context more than less intense stressors such as water deficit. Overall, the importance of the intensity of historical stress has been underexplored. If persistent stress legacies reflect microbiome shifts to alternative stable states, then following intense enough climate change‐related stress, microbiomes could shift to alternative states with diminished or even the breakdown of function. These alternative states could include mutualism loss and mutualism reorganization (i.e. partner switching or shifts to less beneficial or antagonistic relationships) (Kiers *et al*., 2010). While extreme enough historical stress theoretically can exceed the adaptive ability of microbiomes and lead to the breakdown of plant‐microbial mutualisms, more empirical evidence is needed to demonstrate this in the context of stress legacies. Another important aspect of historical stress is stressor identity, including how historical stressors interact with contemporary stressors of a different type. Potentially, microbiomes with a legacy of one type of stress, such as those that have experienced heat shocks, may become specialized in recovering from it and therefore be more vulnerable to a novel contemporary stressor, such as cold shocks (Jurburg *et al*., 2017). However, this pattern may differ by contemporary stress type; for instance, in one study, microbial functional stability under heat stress, but not under drying‐rewetting, was greater if previously exposed to copper contamination (Tobor‐Kapłon *et al*., 2006). The question arises of why the identity of historical and contemporary stress matters and what factors determine this. Approaches clarifying how different properties of historical stress such as duration, intensity, and identity induce functional shifts at the individual microbe or gene and gene expression levels may help explain these patterns and allow for more generalizable conclusions about stress legacies. Ultimately, understanding how microbial stress legacies shape plant–microbe interactions via altered community composition, function, trait selection, and plant performance would allow us to better leverage them in climate adaptation strategies.

### 4. Plant–soil feedbacks in a changing world

**Fig. 6:**
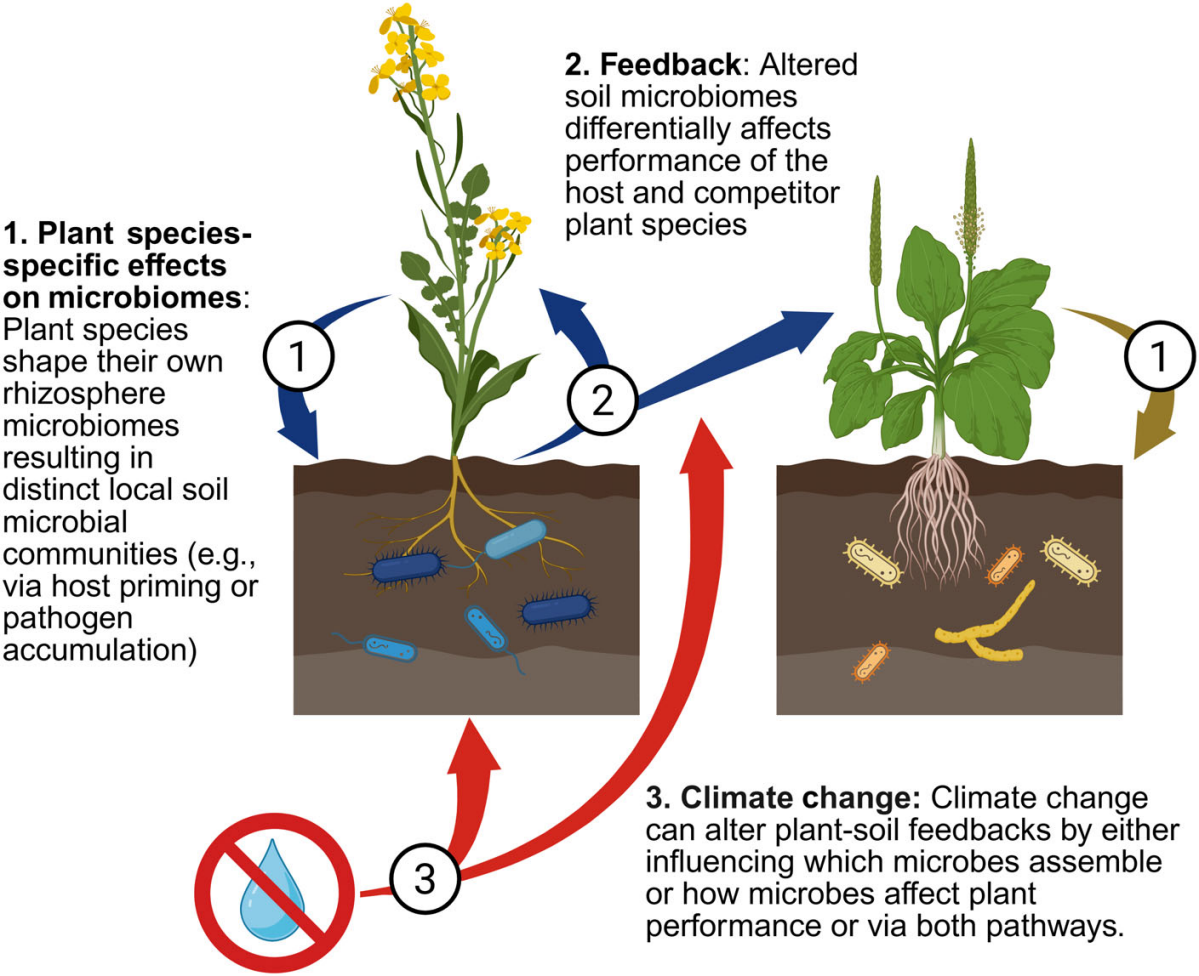
Plant–soil microbiome feedbacks. (1) Plants have species‐specific effects on rhizosphere microbiomes. The first step of plant–soil feedbacks is that each plant species shapes its own rhizosphere microbiome, resulting in distinct local soil microbial communities. This can occur due to active host priming, differences in plant resources available to microbes, or pathogen accumulation from pathogens tracking their hosts. (2) The plant species‐specific changes in the microbiome (i.e. the legacy of a particular plant species) can feedback on the performance of both plant species. Altered species‐specific soil microbiomes differentially affect the performance of their original host species (conspecific plants) and other plant species (heterospecific plants). Here, the arrows in step 2 show the feedback of how the changes that the first plant species (left side) caused in its microbiome (blue microbes) can affect the performance of both the conspecific (left) and heterospecific (right) plant species. (3) Climate change can alter plant–soil feedbacks by either influencing which microbes assemble or how microbes affect plant performance or via both pathways. Here, red arrows highlight both pathways through which climate factors, such as drought (struck‐through water drop), can influence the outcome of plant–soil feedbacks (created in BioRender. Afkhami, M. (2025) https://BioRender.com/i3or6l1).

Similar to the abiotic stress legacy effects on microbiomes (described in the previous section), biotic legacies can be important for understanding plant–microbe interactions and are likely to inform how communities respond to climate change. Of particular interest are plant–soil feedbacks (PSFs) – when plant species' soil microbial communities have different effects on original host plant species (conspecific plants) and other plant species (heterospecific plants) (Fig. 6; Box 1). Plant species differ in the quantity and quality of resources available to microbes, which causes the accumulation of plant species‐specific microbiomes, and these microbial ‘signatures’ can create environmental biotic legacies that influence competing plant species (Kulmatiski *et al*., 2008; Crawford *et al*., 2019). PSFs are measured by comparing the relative performance of plants grown in conspecific‐ and heterospecific‐conditioned microbial communities (i.e. microbial communities with different plant species legacies) (Bever *et al*., 1997). Most plant species generate negative PSFs (i.e. plants perform relatively worse in conspecific soils), likely through the accumulation of plant species‐specific pathogens, which can stabilize plant species coexistence through negative conspecific density dependence (Bever *et al*., 2015). By contrast, positive PSFs (i.e. plants perform relatively better in conspecific soils) can arise when plant priming facilitates the accumulation of microbial mutualists that match the plant's species‐specific needs and destabilize plant species coexistence through positive density dependence. In addition to predicting whether microbes promote or erode plant diversity (Bever *et al*., 1997), PSFs can at least partially explain plant species invasions, successional dynamics, and species abundance distributions (Kardol *et al*., 2006; Mangan *et al*., 2010; Kuebbing *et al*., 2015). Therefore, understanding how PSFs respond to climate change may help predict climate‐mediated shifts in plant community composition.

PSFs are sensitive to changes in climate that can affect which microbial taxa are available to assemble on and around plant roots and the outcomes of plant–microbe interactions (Fig. 6). Climate‐mediated changes in microbial community assembly may be especially impactful if microbial functional groups (e.g. mutualists and pathogens) are differentially affected by climate (Boyle *et al*., 2024). For example, increased aridity is predicted to cause declines in pathogen abundance (Martínez‐Arias *et al*., 2022), driving more positive (coexistence destabilizing) PSFs. However, results thus far are equivocal. While some experiments have found that drier conditions promote more positive PSFs (Rutten & Gómez‐Aparicio, 2018; Milici *et al*., 2025), others have found that drier conditions promote more negative PSFs (Dudenhöffer *et al*., 2022; de Vries *et al*., 2023). A potential explanation for these differences may be whether climate was manipulated only during soil conditioning (influencing microbial community assembly but not directly influencing the outcome of plant–microbe interactions) or during both soil conditioning and PSFs (directly influencing both microbial community assembly and the outcome of plant–microbe interactions). Another potential explanation is that stress legacies (discussed above) may influence how PSFs respond to drought. Thus far, no studies have clearly linked specific changes in microbial community structure or function to climate‐induced changes in feedback. However, climate change can also shift plant–microbe interactions, absent a change in microbial composition. For instance, a plant pathogen differentially affected six plant species under wetter conditions, but species specificity was lost under drought conditions, suggesting that pathogens may play less of a role in determining plant community dynamics during droughts (Joachin *et al*., 2023).

There are many promising avenues for future work on climate change and PSFs. First, novel experimental designs and tools may help unpack the factors that underlie context dependency in PSF responses to climate. For example, the incorporation of molecular tools, such as transcriptomics and genomics (discussed in the Integrating Molecular Mechanistic Knowledge section), can help elucidate which microbial functions are active during PSFs under climate stress (e.g. integrating PSF into a study like Revillini *et al*., 2025, which used manipulative experiments and transcriptomics to evaluate how fire affects the regulation of soil microbial pathways involved in carbon metabolism, nutrient cycling, and other important functions). Second, more explicitly incorporating microbial dynamics, such as successional shifts, inter‐microbial interactions, functional redundancy, and complementarity, into our understanding of PSFs may help refine predictions for how PSFs will respond to changing climates. Third, although climate variables can alter the magnitude and even direction of PSFs, PSFs could alternatively influence the resilience of plant‐microbial interactions to climate change, but the detection of PSFs in resilience would require studies directly manipulating PSFs *before* climate disruption (Rudgers *et al*., 2025). Finally, both plants and microbes are sensitive to axes of climate change other than mean precipitation (e.g. Warneke *et al*., 2023), so incorporating those responses in PSF experiments may be critical for making predictions of plant community change under future climates. Experiments manipulating multiple attributes of climate change (i.e. drought, fire frequency, salinity) both singly and in different combinations could provide important insight into PSF responses to the full range of complex stress these organisms experience.

### 5. Integrating molecular mechanistic knowledge of climate change effects

Understanding mechanisms can meaningfully increase our ability to predict, and sometimes even mitigate, the effects of climate change (Fuller *et al*., 2016). Typically, when climate change research moves from describing patterns to identifying underlying causes, the focus is on ‘ecological mechanisms’, including much of what has been discussed throughout this paper. By contrast, research into climate change and *molecular* mechanisms (Box 1) of microbial mutualisms has historically remained siloed with little crosstalk between subdisciplines. Because microbes' small size necessitates navigating, responding to, and regulating their environments almost exclusively at a molecular scale, the distinctions between ecological and molecular mechanisms for microbes often blur. For instance, density dependence and autoregulation of mycorrhization in AM symbiosis – where colonization is inhibited when already high – represent an ecological and a molecular perspective of the same regulatory process. Thus, exclusion of molecular mechanisms is likely hindering our ability to holistically understand the underlying causes of how climate change affects plant–microbe interactions. Research bridging this gap may improve our ability to predict when climate change will disrupt plant‐microbial mutualisms (Rudgers *et al*., 2020) and help improve resilience in natural and agricultural ecosystems (Ge & Wang, 2025).

Recent sequencing and computational revolutions have opened new possibilities for understanding mechanisms underlying climate change effects on biodiversity and ecosystem function at all levels of biological organization, including at the molecular and genetic levels. For example, metatranscriptomic studies have shown that higher intensity wildfires can enrich bacterial genes involved in heat resistance, fast growth, and pyrogenic carbon utilization (Nelson *et al*., 2022). While new sequencing toolkits have led to vast amounts of molecular data that can now be paired with bulk measurement of microbial function, the field often still lacks overarching frameworks for integrating these findings across environments and predicting the effects of interacting global change factors on plant–microbe systems. Broad‐scale, metagenomic analyses demonstrate that microbial communities adapt both ecologically (e.g. shifts in composition) and evolutionarily (e.g. spread of mutations) to chronic and acute environmental stress by enriching for ameliorative and stress‐tolerant gene functions (Knight *et al*., 2024; Zhang *et al*., 2024). These adaptations can create stress legacies in microbes (discussed above) that enhance plant tolerance to stressors like warming and drought (Allsup *et al*., 2023). While the pace of microbial adaptation may not match the rate of global change, its occurrence and its buffering effect on plant hosts are clear.

**Fig. 7:**
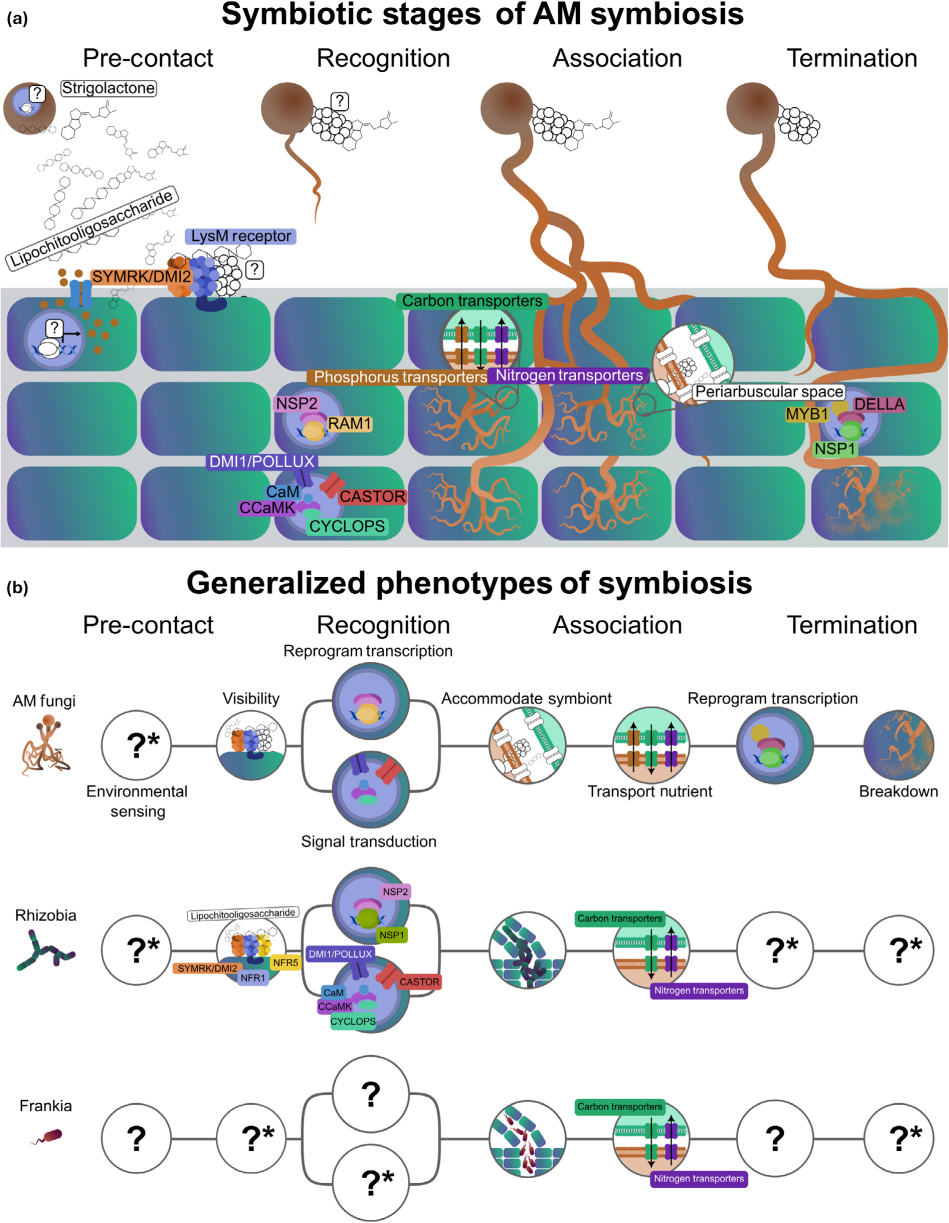
Mechanistic understanding of the stages of beneficial microbial symbiosis with host plants. (a) An example of mechanistic knowledge of these stages in the widespread and vital symbiosis between plants and arbuscular mycorrhizal fungi. Integrating this information could be important for understanding climate change–induced disruption of this crucial symbiosis. During pre‐contact, mycorrhizal fungi and plants release diffusible signals (lipochitooligosaccharides and strigolactones, respectively) if environmental conditions are conducive to symbiosis. Then, during recognition, plants and fungi initiate molecular cascades if they recognize each other with transcription factor complexes (RAM1 and NSP2), causing broad changes in gene expression and ion channels mediating nuclear calcium oscillations (e.g. DMI1, CASTOR, and CYCLOPS/CaM/CCaMK complex). These molecular cascades prepare plants for accommodating symbionts. During association, plants and fungi trade nutrients across the symbiotic interface (i.e. periarbuscular membrane) through carbon, nitrogen, and phosphorus transporters. Finally, during termination, plants preferentially degrade arbuscule trading centers in response to transcriptional reprogramming by a transcription factor complex (NSP1/DELLA protein/MYB1), leading to arbuscular degeneration. (b) These molecular processes in the very well‐studied arbuscular mycorrhizal symbiosis in (a) can be mapped to generalizable phenotypes for symbiont–plant interactions which are shown in (b) (please note that for the sake of brevity, we do not show all genes involved in these mechanisms). In mycorrhizal fungal symbioses, when plants sense that the environment is no longer conducive to AM symbiosis (like in high phosphorus conditions), plants reduce their visibility to the symbiont by producing less strigolactone and lipochitooligosaccharide receptors and expedite termination of the symbiosis. These same steps likely occur in other microbial symbioses because environmental sensing, reducing visibility, and quickening termination are effective host control strategies for reducing interactions under unfavorable conditions, regardless of the specific symbiosis. We can glean key lessons about generalizable mechanisms and identify important knowledge gaps in our understanding of mechanisms underpinning specific symbioses through a cross‐system mechanistic framework. In the figure, question marks (?) signify either a lack of or limited information about which molecules are involved. Question marks with asterisks (?*) signify that genes involved in these processes have been identified, but work is ongoing to confirm the generality of these identified genes for regulating these symbioses across plant species.

Large archives of repeatable and broad‐scale molecular information relevant to climate change – such as gene‐level responses to salinity (Ren *et al*., 2018), drought (Xu *et al*., 2021), and warming (Johnston *et al*., 2019) – have emerged in recent years. These rich resources offer novel opportunities to synthesize and explore general principles of climate stress on plant‐microbial interactions, asking broad questions such as the following: (1) can we better predict the outcomes of plant‐microbial interactions under different types of stressors if we understand the molecular basis of how those shifts are occurring?, (2) what genetic and regulatory mechanisms allow for microbes to provide amelioration to plants under rapidly changing climates (e.g. the relative importance of classical genetic inheritance, horizontal gene transfer of plasmids/genomic islands, and DNA methylation)?, (3) are there keystone microbes or keystone microbial functions that underpin resilient plant‐microbial mutualisms?, and (4) are specific gene families consistently enriched or expanded under particular classes of climatic stress across entire microbial communities or within keystone microbes? Further, integrating some of our existing mechanistic understanding of plant‐microbial interactions with experiments related to climate change could yield important new insights and possible mitigation strategies. For example, there is detailed knowledge of the regulatory pathways and underlying genes involved in the different interaction stages of some important mutualisms between plants and beneficial symbionts, like AM fungi. This includes the establishment (i.e. pre‐contact signaling and recognition of symbionts), active association, and termination of the symbiotic relationships (Fig. 7). Targeted investigation of how these pathways and genes respond to different types of climate change stressors could inform *how* climate change disrupts these interactions (e.g. inhibiting expression of pathways involved in pre‐contact communication on the side of the plant or the symbiont, disrupting pathways that maintain trade centers leading to premature termination of relationships). In time, this knowledge could be useful for the translational goal of mitigating disruptions of the symbiosis through targeted breeding for resilient plant pathways, providing supplementary resources at critical stages of the interaction, and so on. Linking molecular data and mechanisms to ecological theory and community/ecosystem outcomes is not only challenging but also invaluable for holistically advancing toward a unified predictive framework and even developing plans to mitigate disruptions to these interactions undergirding biodiversity and ecosystem services.

## IV. Conclusion

Plants and beneficial microbes structure ecosystems worldwide through complex networks of interactions among plants, microbes, and their environments. These complicated, three‐way networks of feedbacks often make the ecological consequences of climate change difficult to predict. However, this burgeoning field has also provided important new insights, which allow us to make specific, realistic predictions under some climate change contexts (e.g. Figs 2, 3, 4, 5, 6). Further, opportunities exist to build a comprehensive, integrative framework – this effort will require significant collaboration across fields, from molecular biology to ecosystem ecology, in order to address the knowledge gaps spotlighted by this review. As this synthesis revealed, outcomes hinge on several factors, including interactive effects of directional change and increased variability in climate stressors, the organization of microbial communities into functionally redundant or complementary groups, and legacy effects of past stress regimes through selection of stress‐tolerant species or for genetic adaptation. To safeguard critical ecosystem services provided by plant and microbial communities from intensifying climate change, we must begin building integrative frameworks that unite ecological theory, mechanistic insight, and real‐world complexity. Throughout this review, we identified key knowledge gaps, synthesized cross‐disciplinary advances, and outlined paths forward to improve our understanding and ability to predict climate change effects on plants, microbes, and the ecosystem services they sustain. By bridging disciplines and highlighting both progress and persistent challenges, we hope this review will catalyze the integrative efforts needed for the complex synthesis required to ensure resilience of plant‐microbial interactions in a rapidly changing world.

## Competing interests

None declared.

## Author contributions

All authors contributed meaningfully to the writing and editing of the review paper. Figures were made by MEA, AHR, VWL and DJH. MEA led and managed the project with help from KMC. All other authors (ATC, CGD, DJH, VWL, AHR, JAR and JRS) contributed equally to the paper and are listed in alphabetical order.

## Disclaimer

The New Phytologist Foundation remains neutral with regard to jurisdictional claims in maps and in any institutional affiliations.

## Supporting information


**Methods S1** Methods on Literature Search for Fig. 1.
**Table S1** Web of Science searches for plant‐microbial interactions with different climate change stressors.
**Table S2** Paper counts from Web of Science searches for plant‐microbial interactions with different climate change stressors.
Please note: Wiley is not responsible for the content or functionality of any Supporting Information supplied by the authors. Any queries (other than missing material) should be directed to the *New Phytologist* Central Office.

## Data Availability

Data used in Fig. 1 graphs are available in the Supporting Information. No additional data were used.
