# Supplementary material for: Unraveling complexity in climate change effects on beneficial plant–microbe interactions: mechanisms, resilience, and future directions
Source: New Phytol. 2025 Oct 14;249(1):93–113. doi: 10.1111/nph.70644 (PMC12676105; doi:10.1111/nph.70644)
Supplement: Supplementary file 1 — Methods S1 Methods on Literature Search for Fig. 1. Table S1 Web of Science searches for plant‐microbial interactions with different climate change stressors. Table S2 Paper counts from Web of Science searches for plant‐microbial interactions with different climate change stressors. Please note: Wiley is not responsible for the content or functionality of any Supporting Information supplied by the authors. Any queries (other than missing material) should be directed to the New Phytologist Central Office. [file NPH-249-93-s001.pdf]

New Phytologist **Supporting Information**

Article title: **Unravelling complexity in climate change effects on beneficial plant–microbe interactions: Mechanisms, resilience, & future directions**
Authors: Michelle E. Afkhami, Aimée T. Classen, Collin G. Dice, Damian J. Hernandez, Vicki W. Li, Amanda H. Rawstern, Jennifer A. Rudgers, John R. Stinchcombe, and Kerri M. Crawford
Article acceptance date: 15 September 2025

The following Supporting Information is available for this article:

Table S1 Web of Science searches for plant-microbial interactions with different climate change stressors

Table S1. Web of Science searches for plant-microbial interactions with different climate change

Climate Change Related Stressor	Web of Science Searches	Papers Counts: All Available (1945-present)	Papers Counts: Last 10 Years (2014-2024)
Drought	plant AND microb* AND drought AND climate change	1068	916
Salinity	plant AND microb* AND salin* AND climate change	337	298
Snow	plant AND microb* AND snow* AND climate change	211	150
Fire	plant AND microb* AND fire AND climate change	168	127
Flood	plant AND microb* AND flood* AND climate change	143	115
Hurricane	plant AND microb* AND hurricane AND climate change	10	7

Table S2 Paper counts from Web of Science searches for plant-microbial interactions with different climate change stressors

Table S2. Paper counts from Web of Science searches for plant-microbial interactions with different climate change

Climate Stressor	Year/Time	# Papers Returned
drought	2014	22
drought	2015	26
drought	2016	39
drought	2017	33
drought	2018	54
drought	2019	61
drought	2020	78
drought	2021	94
drought	2022	145
drought	2023	166
drought	2024	198
drought	10 years	916
drought	all time	1068
fire	2014	1
fire	2015	3
fire	2016	12
fire	2017	7
fire	2018	6
fire	2019	15
fire	2020	16
fire	2021	15
fire	2022	17
fire	2023	21
fire	2024	14
fire	10 years	127
fire	all time	168
flood	2014	1
flood	2015	3
flood	2016	7
flood	2017	8
flood	2018	7
flood	2019	5
flood	2020	12
flood	2021	15
flood	2022	21
flood	2023	13
flood	2024	23
flood	10 years	115
flood	all time	143

hurricane	2014	0
hurricane	2015	0
hurricane	2016	0
hurricane	2017	1
hurricane	2018	0
hurricane	2019	0
hurricane	2020	1
hurricane	2021	2
hurricane	2022	1
hurricane	2023	1
hurricane	2024	1
hurricane	10 years	7
hurricane	all time	10
salinity	2014	7
salinity	2015	5
salinity	2016	4
salinity	2017	7
salinity	2018	14
salinity	2019	15
salinity	2020	28
salinity	2021	28
salinity	2022	51
salinity	2023	51
salinity	2024	88
salinity	10 years	298
salinity	all time	337
snow	2014	11
snow	2015	9
snow	2016	15
snow	2017	15
snow	2018	15
snow	2019	16
snow	2020	15
snow	2021	16
snow	2022	18
snow	2023	13
snow	2024	7
snow	10 years	150
snow	all time	211

*Note that "all time" refers to 1945-present which is all the dates available on Web of Science.

Methods S1: Methods on Literature Search for Figure 1: To gain insight into the possibility of differential study effort among climate change-induced stressors, we determined the total number of publications for each of six climate change-related stressors (i.e., drought, salinity, changes to snow, fire, flooding and hurricanes) at two time scales: recently (2014-2024) and over longer time frames (1945–present; the longest time frame available for the database). We also investigated the percent change in publications rate for each topic over the last decade. The data used in this analysis were collected from Web of Science (Accessed on: May 3, 2025), and all searches used the "Topic" designation for the search terms, which searches the title, abstract, keyword plus, and author keywords. The general form of the search was: plant AND microb* AND climate change AND X where X was the specific climate stressor (see Table S1 for specific strings). For the data on the total number of publications (Table S1-S2), we summed the number of publications across the last decade or across all years that met the search criteria. To obtain the rate of change results, we regressed the paper counts on year for each climate factor across the last decade (2014–2024; Table S2). Prior to regression, we transformed the number of papers in each year for a given climate factor ($\log_2$ transformation) to improve data normality for the analysis. We calculated the % change in the publication rate per year for Figure 1 by backtransforming and converting to percent change. This is done by calculating $(2^{\beta} - 1) \times 100$ where β is the regression coefficient on a log2 scale (where each unit increase would represent a doubling).
